# The mandibular response to occlusal relief using a flat guidance splint

**DOI:** 10.1007/s12548-013-0093-8

**Published:** 2013-09-17

**Authors:** G. Reichardt, Y. Miyakawa, T. Otsuka, S. Sato

**Affiliations:** 1 Private Dental Office „Ihre Zahnärzte“, Landhausstrasse 74, 70190 Stuttgart, Germany; 2 Department of Craniofacial Growth and Developmental Dentistry, Kanagawa Dental University, 82 Inaoka-cho, 238-8580 Yokosuka, Kanagawa Japan

**Keywords:** Sleep bruxism, Tooth grinding, Brux checker, Temporomandibular joint, Temporomandibular disorders, Occlusal splint

## Abstract

**Background:**

The mechanism of action of occlusal splints used for the successful treatment of temporomandibular disorders (TMD) remains unclear and controversial.

**Aim:**

The aim of this study was to observe the mandibular response during sleep bruxism (SB) on the elimination of occlusal influences by using a flat anterior and lateral guidance splint (FGS).

**Material and method:**

Any changes in mandibular movement patterns and condylar position with the introduction of this tool were measured. Current SB activity on the natural dentition was evaluated using a Brux Checker® (BC) and compared with the activity after insertion of an FGS in 153 subjects.

**Result:**

The spatial mandibular position changed individually with a tendency toward forward and downward movement. The insertion of an FGS led to a change in the topographical condyle-fossa relationship and seemed to create an “unloading” condition for the temporomandibular joint. It was found that increased angulation of the maxillar incisors was responsible for altered muscular activity during sleep.

**Conclusion:**

The masticatory organ appears to self-regulate and to provide an oral behavior modification, which may be more physiological using the FGS as a compensating factor. In this context, it is assumed that sleep bruxism in terms of parafunctional activity is a physiological function of the masticatory organ. The results of this study indicate the importance of controlling anterior guidance in the functional reconstruction of human occlusion.

## Introduction

Many health problems related to the masticatory organ and neighboring structures, such as temporomandibular disorders (TMD) are often associated with parafunctional activities. Oral splints are the most common therapeutic approach used to treat patients diagnosed with TMD [1] and protect the teeth from damage resulting from forceful jaw muscle contractions and reduce concomitant orofacial pain if present [2–5]. There is no consensus on the clinical indications and functioning of oral splints. With respect to the placebo effect, changes in occlusion, improvement in jaw muscle function, oral behavior modification, the recruitment of different motor units, behavioral intervention and new positioning of the condyle and/or the articular disc, the mechanism of efficacy is not fully understood [6–11]; however it is generally recognized that splints are useful in the conservative treatment of TMD [6–8, 10, 12]. Regardless of the mode of action, several randomized clinical trials and literature reviews have documented the therapeutic effectiveness of oral splints [13–16]. It is difficult to study sleep bruxism (SB) because of the difficulty in evaluating the actual oral behavior; however, the Brux Checker® (BC) which is an effective tool for diagnosing and classifying these phenomena has been on the market for several years and is available for functional analysis. It is possible to visualize occlusal patterns which are clinical signs of grinding on occlusal interferences in SB [17, 18]. Especially SB is defined as a non-functional activity of masticatory muscles during sleep [19]. There is no evidence that current occlusal interference could be a factor in static and dynamic occlusion leading to muscular hyperactivity and structural defects. Therefore, this was targeted as the topic of this research. The aim of this study was to investigate the effect of the occlusion on oral behavior modification such as parafunction during sleep. The effect of a flat anterior and lateral guidance splint (FGS), which provides an environment free of posterior contact during mandibular translation was analyzed and the behavior of the jaw muscles in relation to changes in the spatial mandibular position that lead to new positioning of the condyles and the modification of oral behavior was evaluated.

## Material and methods

### Subjects

Between January 2009 and December 2010 a total of 153 consecutive patients (62 males and 91 females; average age, 46.6 years, range, 18–77 years) representing TMD with and without subjective symptoms, were enrolled for this study. Condylographic tracing (Cadiax, Gamma Dental, Klosterneuburg, Austria) was conducted and the individual condylar hinge axis of each patient and its casts were transferred to an articulator.

For inclusion in the study the patients had to meet all of the following criteria: (1) a set of permanent teeth in the maxilla and mandible with reliable posterior support on both sides, (2) a complete set of frontal dentition (canine to canine), (3) aged 18–80 years and in good general health. The exclusion criteria were previous experience with occlusal splint therapy, any physiotherapy within the past 6 months, the use of medication with possible effects on muscular behavior, alcohol or drug abuse and ongoing dental therapy including orthodontic treatment.

### Experimental procedure and oral splint fabrication

The study evaluated two oral devices.

**Fig. 1:**
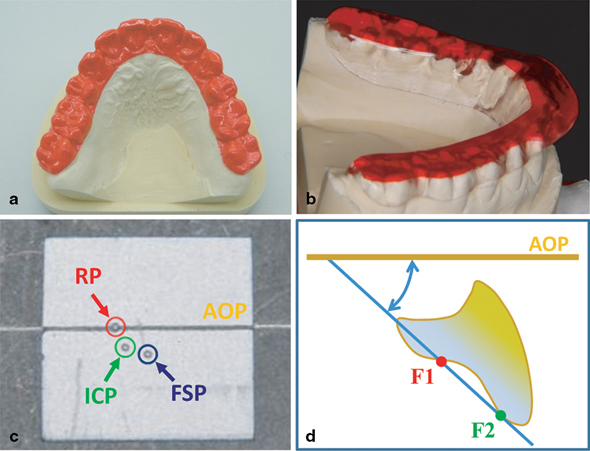
Method of data acquisition and analysis **a** Brux Checker® on a stone model of a patient’s maxillar dentition, **b** a dyed flat guidance splint on a stone model of a patient’s mandibular dentition, **c** spatial mandibular positions and **d** occlusal guidance measurement

(1) A set of BCs was used for the maxilla and the mandible to observe the SB on the present dentition of the subjects (Fig. 1a).

(2) An FGS covering the teeth with a flat posterior support zone was inserted on the mandibular arch; the angulation of the lateral and anterior guidance was minimal (as flat as possible) but steep enough to disocclude all premolars and molars in any dynamic mandibular activity (Fig. 1b). Maxillary and mandibular arch impressions were obtained with alginate in an individualized tray and models were cast in artificial plaster type 4 dental stone (Fujirock EP, GC, Leuven, Belgium). The centric relation, the reference position (RP) was obtained with a bite plate (light curing custom tray material, Supertec, DMG, Hamburg, Germany) and optimized adaptation to the maxillar dentition was implemented using pattern resin (acrylic resin for patterns, Pattern Resin LS, GC, Tokyo, Japan). The bite registration of the mandibular dentition (3/7) was preserved with Alu-wax (accurate bite registration wax, Alminax, Kemdent, Swindon, UK). A kinematic face bow (Condylograph, Gamma Dental) was used to mount the models in an adjustable articulator (Reference SL, Gamma Dental) with a standardized verticalization of 7 mm on the incisal pin. The BCs (analytic foil 0.1 × 125 mm, Brux Checker®, Scheu Dental Technology, Iserlohn, Germany) were based on the two models and each was used for two nights separately in the maxilla and mandible according to previous reports [17]. The FGS (cold curing denture base material, Pro Base Cold, Ivoclar, Schaan, Liechtenstein) was fabricated using a wax-up method and pressed on the mandibular model. It was inserted and adjusted in the patient’s mouth without any manual guidance by the operator while the patient was placed in a sitting position in a dental chair. Uniform occlusal contacts were created to hold the Shimstock foil (Shimstock foil 8 μ, Hanel, Langenau, Germany) on the lingual cusps of the molars and the premolars against the splint to provide reliable static stability. The anterior teeth and the cuspids were lightly touching the splint but not holding the Shimstock foil. Lateral movements were guided by the tips of the maxillar canines; protrusive movements were guided by the incisal edges of the maxillar front teeth against a horizontal anterior ramp of the FGS. In any excursion, dynamic interference in the molars and the premolars was eliminated. The same operator (GR) provided the treatment and each patient was given the same instructions. The patients were asked to wear the splint continuously for 24 h except when eating and when cleaning the teeth. After 7 days in the following session, the splint was adjusted using the described adjustment procedure. To obtain reproducible stability for the FGS with no change from one weekly appointment to the next 2–5 sessions were necessary.

### Recording the static mandibular position and dynamic activity during sleep

To visualize the muscular reaction and static stability of the mandible and the dynamic activity on the FGS the color indicator effect of red dye (Acid red 51, Morimoto Chemical, Tokyo, Japan) was used. Subjects wore the red dye treated splint for two consecutive nights. The following morning digital photographs (digital camera, D90, Nikon, Tokyo, Japan; dental lens system, DCN16-LV/GP2, Sonic Techno, Tokyo, Japan) of the result were obtained and four Alu-wax stops (3, 3 and 7, 7) were placed on the splint. The splint was reinserted in the subject’s mouth and the mouth was closed normally. The acquired and conserved mandibular position, which was labeled as the occlusion relieved position (ORP), was retransferred to the articulator using the splint with the four Alu-wax stops and the original casts. To evaluate the effect of an FGS, three reliable spatial mandibular positions were compared (Fig. 1c):

1. Reference position (RP)
2. Intercuspal position (ICP)
3. Occlusion relieved position (ORP)

Comparative measurements of the patient casts were performed using the Condylar Position Measurement (CPM SL, Gamma Dental). Each spatial mandibular change was accurately measured with a magnifying glass (# 2004 Scale Loupe 10x, PEAK, Tokyo, Japan).

### Classification of the result between Brux Checker® and flat guidance splint

The major axis of the wear facets of the canines and central incisor found on BC and FGS was measured using a magnifying glass and the values were mapped in relationship to one another. A matching type group to a coincidence less than 30 % was defined. Higher values were defined as changing type group.

### Measurement of occlusal guidance

To investigate the influence of occlusion on parafunctional activities, the occlusal guiding surfaces to the AOP in the maxilla were measured according to previous reports [20] (3d- Digitizer, Gamma Dental) (Fig. 1d).

### Statistical analysis

A Wilcoxon signed rank test was used to compare the spatial distance obtained from RP-ICP with the RP-ORP provided by the FGS. The significance level was set at *p* < 0.05. Student’s paired t-test was used to compare the mean angle of each tooth between the matching type and the changing type. The significance level was set at *p* < 0.05.

## Results

### Reactive adaptation of the condylar position

**Fig. 2:**
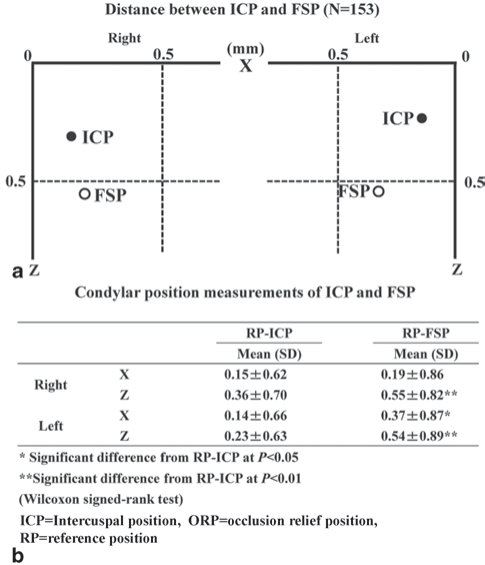
The changes in spatial condylar relationships. **a** The average sagittal condylar change of ICP and ORP compared to RP (=0) and **b** the mean SD of the spatial change of ICP and ORP compared to RP in the x and z direction. No significance is shown by Δy

The ORP had a large standard deviation. The average tendency was downward and forward compared with the RP and the ICP (Fig. 2).

### Classification of grinding pattern on the dentition (Brux Checker analysis)

**Fig. 3:**
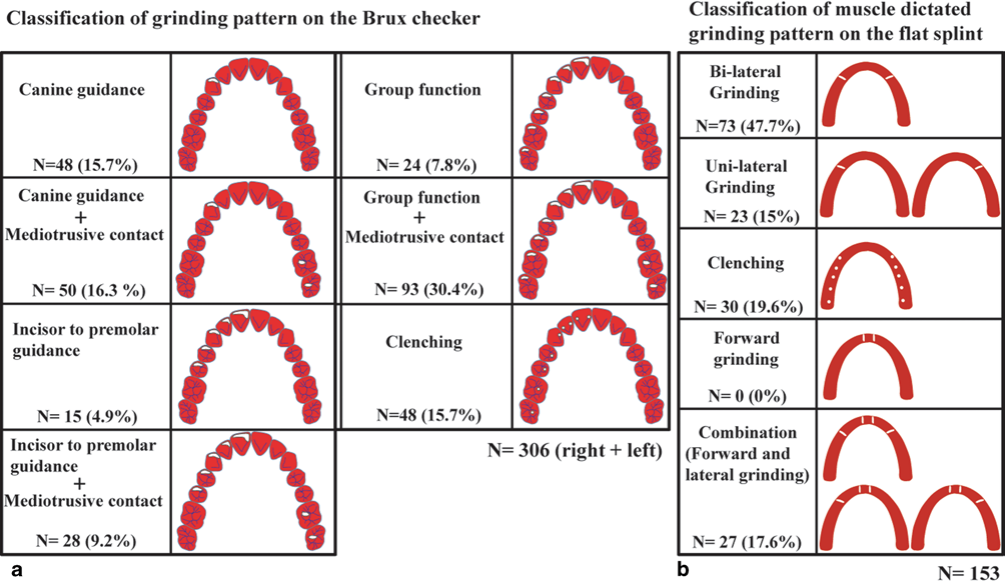
The classification of grinding activities in sleep bruxism and their distribution. **a** The grinding pattern distribution in natural dentition observed using a bruxchecker and **b** the distribution of occlusion relieved and muscle-dictated grinding found after application of the flat guidance splint. The white sites indicate the bruxism activity

After wearing the BC for two consecutive nights, the grinding pattern of the subjects had transferred to the surface of the BC and was observed by the removal of the painted areas, which were removed by clenching and grinding. The wear site and pattern were then evaluated. The BC presented different characters in SB that were classified into seven groups (Fig. 3a).

### Classification of the grinding pattern on the flat guidance splint

The FGS results were classified into five types in a manner similar to the BC (Fig. 3b).

### Characteristic of sleep bruxism behavior on dentition and flat guidance splint

**Fig. 4:**
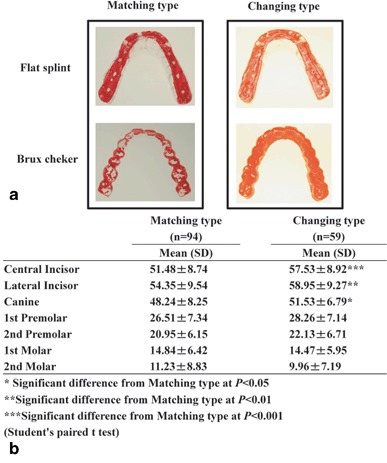
Examples of the mandibular response to occlusal relief using a flat guidance splint and the corresponding statistical distribution. **a** Clinical examples of the matching and the changing types and **b** the influence of steep anterior guidance in the maxillar dentition

Comparing the SB behavior on the dentition with the use of an FGS, a change in the character of the behavior was observed in many subjects. One group showed the same direction of mandibular movement for both conditions (matching type). The other group showed a different activity (changing type) and these groups were distributed 94:59 (Fig. 4a).

### Influence of occlusal guidance on oral behavior

The occlusal guidance of all maxillae was measured to investigate its influence on parafunctional activity. The result of the BC reflects the behavior in present dentition. The mandibular incisors and cuspids glide along the surfaces of their maxillar counterparts and create individual wear facets. Using an FGS, any posterior contact during mandibular translation was eliminated; the effect of the anterior “flat” ramp provided an immediate anterior and lateral guidance, less accentuated as individually possible because only the tips of the maxillar incisors and canines slightly touched it. By this means a pure canine guidance was installed which was kept as shallow as possible. The predominant factor in the changing group was the increasing angle of the central (*p* < 0.001) and lateral incisors (*p* < 0.01) of the maxilla. The canines also demonstrated a significant difference (*p* < 0.05). For the other teeth, the difference in angulation between the two types was not significant.

## Discussion

The objective of this study was to analyze the effect of an FGS on oral behavior modification such as parafunction during sleep. This study is probably the first to analyze the changes in muscle behavior caused by occlusion relief, the elimination of present occlusion as an influencing factor for the neuromuscular system. A previous study with young healthy subjects showed that an increase in the steepness of occlusal guidance leads to a modification of masticatory muscle behavior and condylar position [21] and may contribute to the development of TMD symptoms. These results were supported by modern functional magnetic resonance imaging (fMRI) studies. It was demonstrated that a steepened frontal guidance creates a retral forced bite that leads to a compressive condition in the TMJ accompanied by increased activity in the stress- assimilating areas of the brain [22, 23]. In this study the opposite approach was used to show how muscles can be influenced by anterior and lateral guidance. Because an FGS eliminates all occlusal interferences that may disturb harmonic mandibular function, the end results of the study show a spatial change in the condylar position, which varied among individuals. However, it is arguable whether the difference in condylar position translates into joint load reduction. The data indicate a slight ventral and caudal shift of the condyle in subjects wearing an FGS. However, the high reproducibility of ORP may indicate that the masticatory organ experiences some sort of muscular self-regulation. These results should be supported by controlled fMRI studies that investigate brain activity and could be the focus of further studies. Using the BC technique the actual SB activity of each subject was investigated. The results are consistent with the results of previous studies suggesting the existence of a classifiable effect of grinding on the dentition due to SB [16, 17]. In this sample a pure clenching group was additionally observed and added to the previous classifications. The effect of occlusion relief using the FGS was investigated and the results indicate different but classifiable grinding patterns that could be divided into five groups. By comparing the wear sites (paint removal caused by SB) as well on the BC as on the FGS, many subjects showed changes in quantity and direction. It can be assumed that these changes of parafunctional activity are due to a modification of the neuromuscular system. One group demonstrated coincidence of mandibular movement under both conditions (matching type); the other group demonstrated a different characteristic (changing type) and a ratio of 3:2 was observed for the number of subjects in the groups. To determine the trigger for the change in behavior after occlusion relief, the occlusal guidance of the maxilla was measured to investigate its influence on SB activity. The predominant factor in the changing group was the increased angulation of the maxillar incisors and the cuspids. Other teeth showed no significant influence on muscle behavior. As described earlier steep guidance in the maxillar front teeth may indicate a risk for occlusal trauma [24]; however, the observation of a change in the muscle behavior following splint therapy must be interpreted with caution. The findings of the condition with occlusion relief were taken from an artificial occlusion generated by a splint. Although this technique is currently the conventional method to treat TMD patients, it must be noted that the real effect of splint therapy is still unknown and highly controversial. In conclusion, the mandibular response to occlusal relief using an FGS presented a reliable and stable spatial mandibular position. Occlusion seems to affect muscle activity and oral behavior. Additionally, the steepness of the anterior guidance, particularly the inclination of the incisors, appears to significantly influence muscle activity during sleep. The results of this study support the interest to control anterior and lateral guidance in the functional reconstruction of human occlusion.

### Acknowledgments

This study was performed in the private dental office “Ihre Zahnärzte Landhausstrasse 74” in Stuttgart, Germany, in association with the Kanagawa Dental University Research Institute of Occlusion Medicine, Yokosuka, Kanagawa, Japan. We thank Dr. Christina Rijpstra and Dr. Alain Landry for their encouragement and expert guidance. The authors declare no potential conflicts of interest with respect to the authorship and/or publication of this article.
